# Car@PLGA-NPs target gut microbiota-ER stress axis to combat diabetes

**DOI:** 10.3389/fcimb.2025.1667023

**Published:** 2026-01-09

**Authors:** Wei Zhao, Li Chen, Jing Qing, Zhujia Zhao, Lijuan Xiong, Pingzhen Tong, Ziruo Huang, Yunzhi Chen

**Affiliations:** 1 Department of Endocrinology, The Second Affiliated Hospital of Guizhou University of Traditional Chinese Medicine, Guizhou, Guiyang, China; 2 Department of Preventive Health Care, The Second Affiliated Hospital of Guizhou University of Traditional Chinese Medicine, Guizhou, Guiyang, China; 3 The Second Clinical Medical School of Guizhou University of Traditional Chinese Medicine, Guizhou, Guiyang, China; 4 Clinical Laboratory, The Second Affiliated Hospital of Guizhou University of Traditional Chinese Medicine, Guizhou, Guiyang, China; 5 Department of Pathology, The Second Affiliated Hospital of Guizhou University of Traditional Chinese Medicine, Guizhou, Guiyang, China; 6 Department of Traditional Chinese Medicine Diagnostic Studies, School of basic medicine. Guizhou University of Traditional Chinese Medicine, Guizhou, Guiyang, China

**Keywords:** carvacrol, endoplasmic reticulum stress, gut microbiota, islet, nanoparticle

## Abstract

**Background:**

Previous studies have demonstrated that carvacrol (Car) ameliorates vascular and hepatic injury in db/db mice, but its low bioavailability limits clinical translation.

**Methods:**

To address this, this study constructed carvacrol-loaded polymeric nanoparticles (Car@PLGA-NPs) to enhance carvacrol bioavailability and fully explore its novel mechanisms of action on islet function and gut homeostasis in a diabetic model. We used C57BL/6J db/db mice to measure serum fasting blood glucose, oral glucose tolerance (OGTT), insulin tolerance (ITT), and lipid profiles. Fecal samples were collected for 16S rRNA sequencing to analyze gut microbiota composition and its correlation with host indices. Pancreatic and intestinal tissues underwent histopathological staining, immunofluorescence, and Western blotting to detect endoplasmic reticulum (ER) stress-related protein expression levels (p-IRE1α, XBP1S, PERK, p-ElF2α).

**Results:**

Results demonstrated that Car@PLGA-NPs, compared to free carvacrol, significantly improved insulin sensitivity, reduced fasting blood glucose, ameliorated dyslipidemia, attenuated inflammation, and mitigated oxidative stress in db/db mice. 16S rRNA sequencing revealed that Car@PLGA-NPs remodeled the gut microbiota composition, with *Alloprevotella* abundance showing a negative correlation with colonic ER stress proteins (p-IRE1α and p-ElF2α). Immunofluorescence and Western blotting further confirmed that Car@PLGA-NPs significantly suppressed the expression of ER stress-related proteins (p-IRE1α, XBP1S, PERK, p-ElF2α) in both islet and colonic tissues, demonstrating superior efficacy to free carvacrol.

**Conclusions:**

Collectively, this study confirms that the PLGA nanocarrier effectively enhances carvacrol bioavailability. Car@PLGA-NPs improve islet function and intestinal homeostasis in diabetic mice by remodeling the gut microbiota and subsequently inhibiting ER stress in pancreatic and intestinal tissues, providing a novel nano-drug delivery system and a “microbiota-ER stress” regulatory axis for diabetes treatment.

## 1 Introduction

Type 2 diabetes mellitus (T2DM) is a metabolic disorder primarily characterized by persistent hyperglycemia due to aberrant insulin secretion and/or islet dysfunction (Saeedi et al., 2019; Lin et al., 2024). This condition involves a constellation of pathogenic events, including impaired insulin secretion from β-cells, uninhibited glucagon release from α-cells, a raised α/β-cell ratio, and the accumulation of islet amyloid deposits (Mizukami and Kudoh, 2022). Although dietary control, physical exercise, and oral hypoglycemic agents can effectively lower blood glucose levels, they often inadequately prevent disease progression and associated complications. Therefore, the identification of novel therapeutic targets or agents for treating T2DM and its complications remains an urgent priority.

Given its central role in protein synthesis and modification, homeostasis of the widely distributed endoplasmic reticulum (ER) is crucial for normal insulin production (Renzi et al., 2024), with substantial evidence linking ER dysfunction to the aggravation of type 2 diabetes (Meyerovich et al., 2016). Multiple studies have established that the modulation of ER stress proteins effectively ameliorates diabetic complications. For instance, the suppression of key stress sensors, including X-box binding protein 1 spliced (XBP-1s), phosphorylated inositol-requiring enzyme 1α (p-IRE1α), phosphorylated PKR-like endoplasmic reticulum kinase (p-PERK), PERK, and ElF2α attenuates ER stress and inflammation, thereby improving renal function and pathology in diabetic models (Chen et al., 2016; Shi et al., 2024). Inhibition of granzyme B downregulates key ER stress-related signaling molecules (PERK, p-PERK, ElF2α, p-ElF2α), which in turn mitigates diabetes-associated cognitive impairment (Yang et al., 2024; Zhang et al., 2024). Collectively, these findings indicate that indicate that the dynamics of ER-related protein expression not only mirror the progression of diabetes but also highlight the targeting of ER stress as a viable therapeutic strategy.

Significant differences in gut microbial composition exist between diabetic patients and healthy controls, marked by the notable depletion of several bacterial taxa, such as Firmicutes, Archaea, and *Paenibacillus*. Conversely, *Bacteroides vulgatus*, the *CAG207* phage genus, and Acidaminococcales show therapeutic potential for type 2 diabetes (Wang et al., 2019, Wang et al., 2020). The gut microbiota profile in patients with T2D is characterized by an increased abundance of opportunistic pathogens, a reduced abundance of butyrate-producing bacteria (a key short-chain fatty acid regulating gastrointestinal function), and an elevated Bacteroidetes to Firmicutes ratio (Zhou et al., 2022). Evidence indicates that the inhibition of intestinal IRE1α curtails lipid absorption, which in turn regulates plasma lipids and confers protection against hyperlipidemia-related diseases, including diabetes and metabolic syndrome (Iqbal et al., 2021). Furthermore, trimethylamine N-oxide (TMAO) ameliorates diabetes progression through the dual modulation of the PERK/ElF2α signaling pathway and gut microbiota composition (Govindarajulu et al., 2020). Emerging evidence suggests a crosstalk between the gut microbiota and ER stress in peripheral tissues, forming a ‘gut-pancreas axis’ that contributes to diabetic pathology. However, therapeutic strategies simultaneously targeting both arenas are limited.” Carvacrol, a phenolic monoterpenoid found in various essential oils, has shown promise due to its diverse therapeutic properties, including potential applications in diabetes prevention and antimicrobial activity (Hoca et al., 2024) (Silva et al., 2018). Sun et al. have demonstrated that carvacrol improves blood lipid and glucose levels in type 2 diabetic rats (Sun et al., 2024).We have previously shown that carvacrol can improve blood glucose and insulin resistance in T2DM mice, and has a protective effect on the liver, suggesting that this protective mechanism is related to the regulation of TLR4/NF-κB and AKT1/mTOR signaling pathways (Zhao et al., 2021). Despite its promising therapeutic efficacy in these models, carvacrol suffers from low bioavailability. To overcome this limitation and to probe the interconnected roles of microbiota and ER stress, we developed Car@PLGA-NPs to enhance carvacrol bioavailability and utility, fully explore its potential to improve pancreatic and intestinal function in diabetic mice via modulation of gut microbiota and endoplasmic reticulum stress.

## 2 Materials and methods

### 2.1 Preparation and characterization of Carvacrol-Loaded Poly (DL-lactide-co-glycolide) (PLGA) Nanoparticles (Car@PLGA-NPs)

The nanoparticles were prepared using a modified emulsion-solvent evaporation method based on a previous study (Haque et al., 2018). Briefly, a 3% (w/v) polyvinyl alcohol (PVA) solution was prepared by dissolving 3 g of PVA in 100 mL of ultrapure water under continuous stirring at 60 °C, followed by cooling and storage at 4 °C. Next, 200 mg of PLGA20K-COOH and 1 mL of carvacrol were dissolved in 5 mL of chloroform with stirring for 30 min. This organic phase was then slowly added to 20 mL of the 3% PVA solution, and the mixture was emulsified by sonication (300 W) for 25 min to form a stable milky suspension. Subsequently, 50 mL of ultrapure water was added, and the suspension was stirred magnetically (300 rpm) at room temperature for 1.5 h to evaporate the organic solvent. The resulting suspension was dialyzed extensively against water (with over 5 water changes) for approximately 48 h to remove unencapsulated carvacrol and free PVA. Finally, the suspension was pre-frozen at -80 °C for 6 h and then lyophilized under vacuum for 24 h to obtain a white, porous nanoparticulate powder, which was stored sealed and protected from light at 4 °C. The particle size and zeta potential of Car@PLGA-NPs and empty PLGA nanoparticles (PLGA-NPs) were determined by dynamic light scattering (DLS) using a Zetasizer Ultra Particle Size Analyzer (Malvern Panalytical, Malvern, Worcestershire, UK). The morphologies of Car@PLGA-NPs and PLGA-NPs were determined by transmission electron microscopy (TEM) (FEI Company, Hillsboro, Oregon, USA).

### 2.2 Animals grouping and administration

Male C57BL/6J db/db mice and C57BL/6J db/m+ mice of the same age (7–8 weeks-old) were purchased from Changzhou Kavins Laboratory Animal Co., Ltd. (Changzhou, Jiangsu, China). Mice were housed under controlled conditions (23 ± 3°C, 70 ± 10% humidity) with a 12h light/dark cycle. The approval of animals’ experiments was granted by the Ethics Committee of the Second Affiliated Hospital of Guizhou University of Traditional Chinese Medicine (No.2024101) and the procedures followed were in accordance with institutional guidelines. db/db mice were randomly divided into four groups with db/m+ mice serving as the control group (n =10). The dosing regimen for each group is detailed in **Table 1** (Zhao et al., 2021).

**Table 1:** Experimental group design and dosing regimen.

Group	Treatment (Daily oral)	Duration
Control	equal amount normal saline	6 weeks
db/db	equal amount normal saline	6 weeks
Carvacrol	10 mg/kg	6 weeks
PLGA-NPs	equal amount Car@PLGA-NPs	6 weeks
Car@PLGA-NPs	50mg/ml, 10 mg/kg	6 weeks

Mouse body weight was measured once a week. After a 6-hour fasting period, blood samples from the tail vein was collected from all mice to measure fasting blood glucose (FBG) using a blood glucose monitor (Bayer, Leverkusen, North Rhine-Westphalia, Germany) and was also used as a 0min blood glucose test value. The mice then received an intraperitoneal administration of either glucose solution (1.2 g/kg) or conventional insulin (1 U/kg), and the venous blood of each group was obtained by tail clipped at 15min, 30min, 60min and 120min, respectively, to determine the blood glucose value of each group. It was used to evaluate Oral glucose tolerance test (OGTT) and Insulin tolerance test (ITT). Following the tests, the mice were euthanized via intraperitoneal injection of sodium pentobarbital (200 mg/kg). Blood was collected via orbital bleeding, centrifuged at 3500g, and serum supernatant was obtained. The pancreas and intestinal tract of mice were removed and stored at -80°C for subsequent experiments. Colon segments were collected, rinsed with ice-cold phosphate-buffered saline (PBS) to remove luminal contents and bacterial metabolites, and then stored at -80 °C for subsequent analysis.

### 2.3 Homeostasis model assessment of insulin resistance

Serum insulin levels were measured using an insulin ELISA kit (H203-1-2, Nanjing Jiancheng Bioengineering Institute, Nanjing, Jiangsu, China). The homeostasis model assessment of insulin resistance (HOMA-IR) index was calculated using the formula: HOMA-IR=FBS (mmol/L)×fasting insulin (mU/L)/22.5 to obtain HOMA-IR, to evaluate insulin resistance in mice.

### 2.4 Serum detection

The following mice serum detection kits were purchased from Nanjing Jiancheng Bioengineering Institute (Nanjing, Jiangsu, China), and the corresponding kits are as follows: Insulin Assay Kit (H203-1-2), Low-density lipoprotein cholesterol Assay Kit (LDL-C, A113-1-1), High-density lipoprotein cholesterol Assay Kit (HDL-C, A112-1-1), Total cholesterol assay Kit(TC, A111-1-1), Triglyceride assay Kit (TG, A110-1-1), Interleukin-1β Assay Kit (IL-1β, H002-1-1), Interleukin-6 Assay Kit (IL-6, H007-1-2), Interleuki-10 Assay Kit (IL-10, H009-1-2), Tumor Necrosis Factor-α Assay Kit (TNF-α, H052-1-2).

### 2.5 Colonic tissue staining

Colon tissues were fixed, paraffin-embedded, and sectioned. Sections were then stained with Hematoxylin & Eosin (H&E), Periodic Acid-Schiff (PAS), and Alcian Blue (AB) following standard protocols to assess general histology, goblet cells, and acidic mucins, respectively.

### 2.6 Immunofluorescence staining

The pancreatic tissues were fixed, paraffin-embedded, and sectioned. After xylene dewaxing, gradient ethanol dehydration, high pressure antigen repair, 1% Triton permeabilization and incubation with goat serum blocking solution. Subsequently, the sections were incubated overnight at 4°C with the following primary antibodies: anti-Glucagon (1:400, 67286-1-Ig, proteintech, Wuhan, Hubei, China), anti-INS (1:400, 15848-1-AP, proteintech, China), anti-XBP1S (1:100, 24868-1-AP, proteintech, China), anti-PERK (1:100, 24390-1-AP, proteintech, China), anti-p-IRE1α (1:100, AF7150, Affinity, Liyang, Jiangsu, China), anti-p-ElF2α (1:200, AF3216, Affinity, China). The next day, the sections were incubated at room temperature with corresponding secondary antibodies: Alexa Fluor^®^ 594 Labeled Goat anti-mouse IgG (ZF-0513, ZSGB-BIO, Beijing, China), Alexa Fluor^®^ 488 Labeled Goat Anti-Rabbit IgG (ZF-0511, ZSGB-BIO, Beijing, China). Finally, the sections were mounted with an anti-fade mounting medium containing DAPI for nuclear counterstaining. The staining results were observed under fluorescence microscope and photographed.

### 2.7 MDA and SOD detection

Mice serum was taken for direct detection. According to the instructions for malondialdehyde (MDA) content detection kit (BC0020, Solebol, Beijing, China) and superoxide dismutase (SOD) activity detection kit (BC0175, Solebol, Beijing, China), preparation the reagents for determination. For MDA, the mixture was 100°C for 60min, centrifuged and the supernatant absorbance was read at 532nm and 600nm. For SOD, the mixed solution reacted at 37°C for 30min, and then the absorbance of each sample at 560nm was measured by Microplate Reader. The MDA content and SOD activity were calculated as per the kit instructions.

### 2.8 16S rRNA sequencing

Total genomic DNA was extracted from fecal samples, and the V3-V4 region of 16S rRNA was amplified with primers 5 ‘-CCTAYGGGRBGCASCAG-3’,5 ‘-GGACTACNNGGGTATCTAAT-3’. The amplified products were detected by agarose gel electrophoresis, the gels were collected and purified, and a sequencing library was constructed using the TruSeq^®^ DNA PCR-Free Sample Preparation Kit (Illumina, San Diego, California, USA). The constructed library was quantified by Qubit and Q-PCR. After the library was qualified, NovaSeq6000 was used for PE250 on-machine sequencing. The reads of each sample were spliced with FLASH (Version 1.2. 11, http://ccb.jhu.edu/software/FLASH/) (Magoc and Salzberg, 2011), After the above processing, the obtained Tags need to undergo the removal of chimeric sequences. The Tags sequences were compared with the species annotation databases (Silva database https://www.arb-silva.de/ for 16) to detect chimeric sequences, and the chimeric sequences were finally removed to obtain the final valid data. According to the top 10 abundant species of each sample in different taxonomic classes (phyla, class, order, family, genus, species), the distribution histogram of relative abundance in Perl is plotted by SVG function. QIIME2 software was used to calculate α and β diversity analysis, and a series of statistical analyses such as LEfSe revealed the differentiation of community structure.

### 2.9 Western blot

Protein was extracted from pancreatic and intestinal tissues of mice in each group. Based on the measured protein concentrations, equal amounts of protein were loaded for SDS-PAGE and subsequently transferred onto PVDF membranes. The PVDF membranes were sealed with 5% skim milk for 2h at room temperature followed by an overnight incubation at 4 °C with the primary antibodies:anti-XBP1S (1:1000, 24868-1-AP, proteintech, China), anti-PERK (1:1000, 24390-1-AP, proteintech, China), anti-p-IRE1α (1:1000, AF7150, Affinity, China),anti-p-ElF2α (1:1000, AF3216, Affinity, China) and anti-GAPDH (1:5000, 60004-1-IG, proteintech, China). On the second day, the membranes were incubated with the secondary antiody (1:5000, ZB-2301, ZSGB-BIO, China) at room temperature. change to "On the second day, the membranes were incubated with the secondary antibodies (1:5000, ZB-2301 and ZB-2305, ZSGB-BIO, China) at room temperature. The protein bands were visualized using enhanced chemiluminescence detection reagents (P1050, Applygen Technologies, Beijing, China) and imaged a gel detection analyzer (Invitrogen; Thermo Fisher Scientific, Waltham, Massachusetts, USA).

### 2.10 Statistical analysis

The detected data and the quantized data are expressed as mean ± standard deviation. All statistical analyses were performed using IBM SPSS Statistics 27 software. Intergroup comparisons were conducted using one-way analysis of variance (ANOVA), followed by Bonferroni’s *post hoc* test for multiple comparisons. Spearman correlation analysis was employed. *P* < 0.05 was considered statistically significant.

## 3 Results

### 3.1 Construction of Car@PLGA-NPs and its effects on insulin secretion and blood glucose in diabetic mice

**Figure 1:**
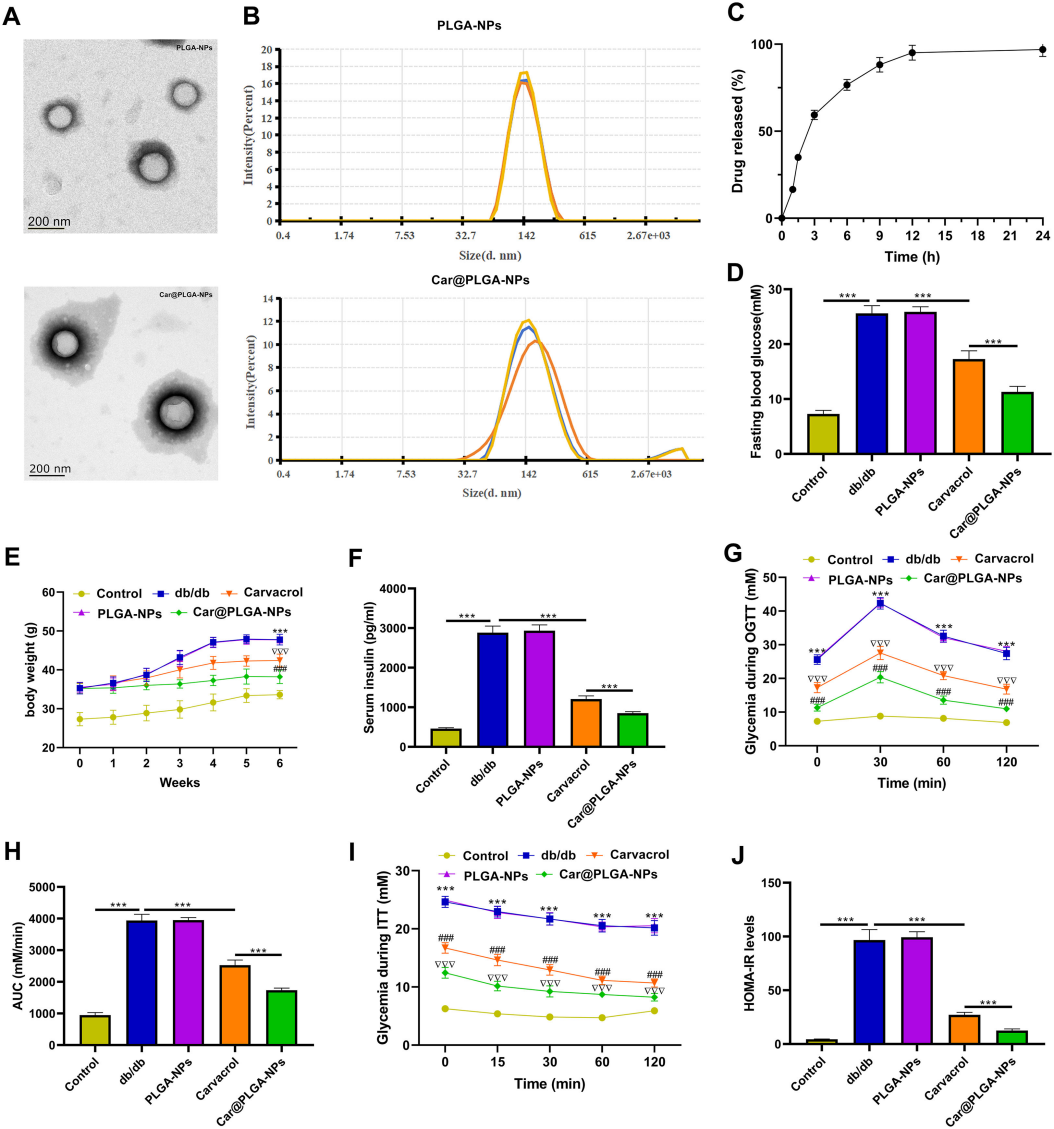
Car@PLGA-NPs improved insulin and blood sugar levels in diabetic mice. **(A)** The morphology of PLGA-NPs and Car@PLGA-NPs was observed under electron microscope. Scale bar, 200nm; **(B)** Particle size distribution of PLGA-NPs and Car@PLGA-NPs; **(C)***in vitro* release behavior of Car@PLGA-NPs presented as the cumulative percentage release; **(D)** Fasting blood glucose of each group after feeding for 6 weeks; **(E)** The changes of body weight in each group after 0–6 weeks of feeding; **(F)** Serum insulin level of each group; **(G)** The change trend of blood glucose in each group after intragastric glucose administration; **(H)** Quantitative OGTT experiment; **(I)** The trend of blood glucose in each group after intraperitoneal injection of insulin; **(J)** Insulin sensitivity was assessed by HOMA-IR. ^***^P < 0.001 vs control group; ^###^*P* < 0.001 vs carvacrol group; ^ΔΔΔ^*P* < 0.001 vs db/db group.

Electron microscopy confirmed the successful synthesis of both Car@PLGA-NPs and PLGA-NPs, demonstrating uniform particle size distribution and spherical morphology (**Figure 1A**). Dynamic Light Scattering (DLS) measurements showed the average size of PLGA-NPs was 128.9 nm, which increased to 139.3 nm upon carvacrol encapsulation, verifying Car@PLGA-NP formation (**Figure 1B**). In the results of the drug release curve study, the drug was released at a relatively fast rate initially. The cumulative release rate reaches 60% after 3 hours and 75% after 6 hours. Then the release rate slows down. After 24 hours, approximately 95% of the drug has been released (**Figure 1C**).

In diabetic (db/db) mice, Car@PLGA-NPs demonstrated superior efficacy compared to free carvacrol. After 6 weeks, fasting blood glucose levels were significantly lower in the Car@PLGA-NPs group than in the carvacrol group (*P* < 0.001, **Figure 1D**). Furthermore, body weight in the Car@PLGA-NPs group remained consistently lower than in the carvacrol group from week 3 onwards (*P* < 0.001, **Figure 1E**). Serum insulin levels were also significantly reduced in db/db mice treated with Car@PLGA-NPs (*P* < 0.001, **Figure 1F**).

Oral Glucose Tolerance Tests (OGTT) revealed consistently lower blood glucose levels in the Car@PLGA-NPs group compared to the carvacrol group (*P* < 0.001, **Figure 1G**). While OGTT-AUC was significantly elevated in untreated db/db mice versus controls, Car@PLGA-NP treatment significantly decreased the AUC (*P* < 0.001, **Figure 1H**). Similarly, Insulin Tolerance Tests (ITT) showed a significant decrease in blood glucose following Car@PLGA-NPs treatment (*P* < 0.001, **Figure 1I**). Critically, the HOMA-IR index (indicating insulin resistance) was significantly lower in the Car@PLGA-NPs group than in the carvacrol group (*P* < 0.001, **Figure 1J**). Collectively, these results demonstrated that Car@PLGA-NPs effectively improved insulin sensitivity and glycemic control in db/db mice, exhibiting significantly greater efficacy than free carvacrol alone.

### 3.2 Effects of Car@PLGA-NPs on Lipid Profile, Inflammation, Oxidative Stress, and Islet Function in Diabetic Mice

**Figure 2:**
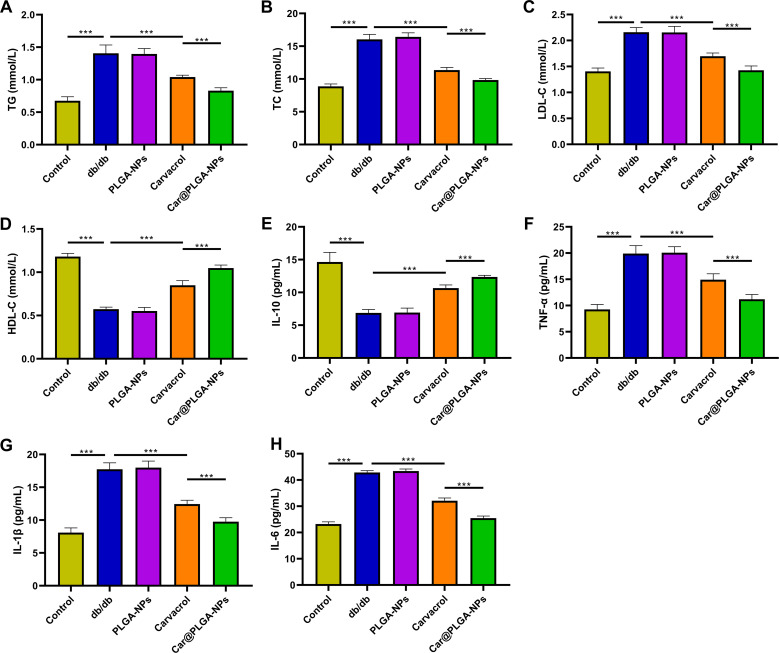
Car@PLGA-NPs improved dyslipidemia, inflammation in diabetic mice. **(A–D)** The contents of TG, TC, LDL-C and HDL-C in serum of mice in each group; **(E–H)** The content of IL-10, IL-1β, IL-6 and TNF-α in serum of mice in each group. ^***^P < 0.001.

**Figure 3:**
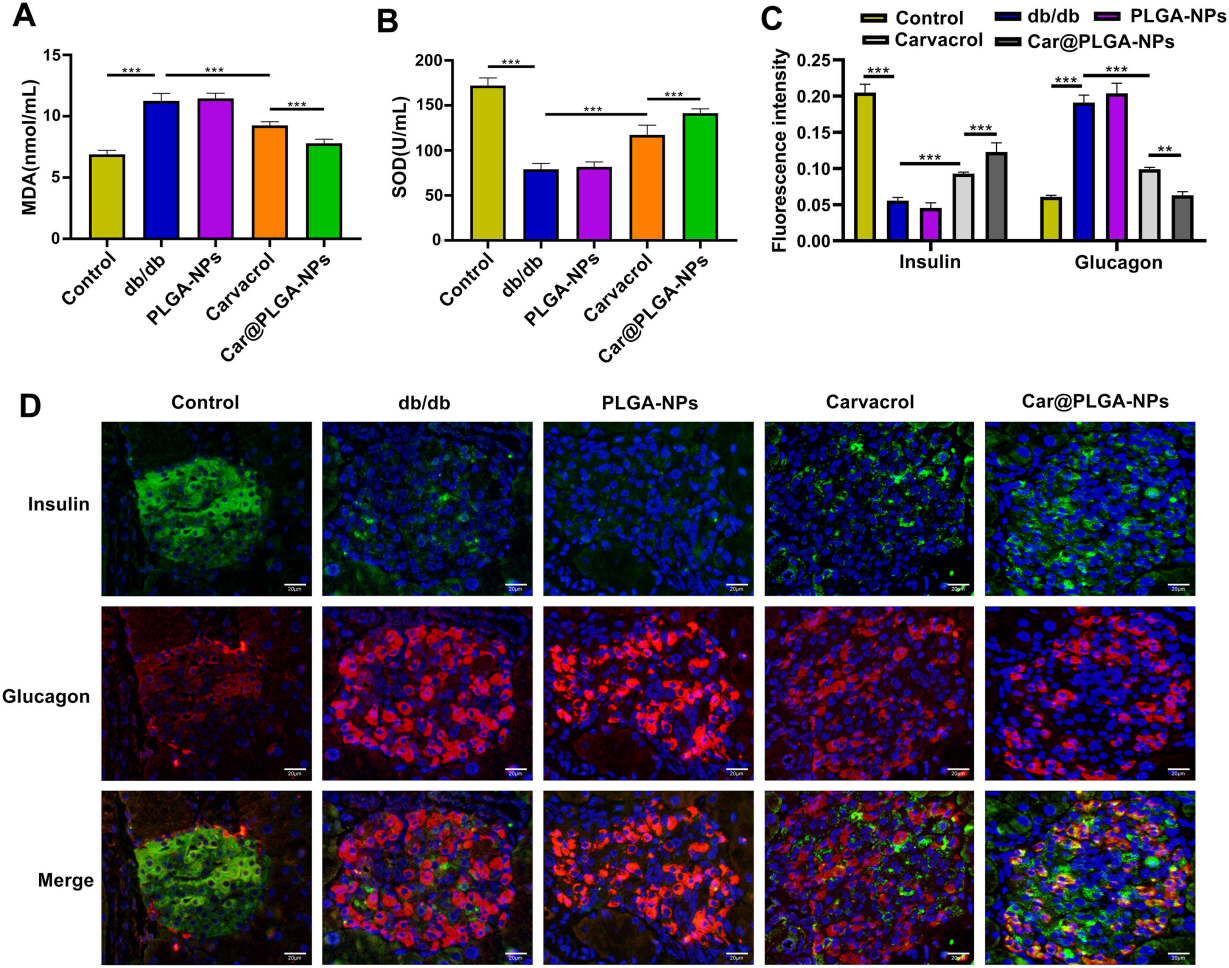
Car@PLGA-NPs improved oxidative stress and islet function in diabetic mice. **(A)** MDA content in serum of mice in each group; **(B)** Serum SOD activity of mice in each group. **(C, D)** Immunofluorescence detection of insulin and glucagon expression in the pancreas, Scale bar, 20μm. ^***^P < 0.001, ^**^P < 0.01.

Car@PLGA-NPs treatment significantly reduced serum levels of LDL-C, TG, and TC compared to free carvacrol alone, while increasing HDL-C levels (*P* < 0.001, **Figures 2A–D**), indicating improved dyslipidemia in diabetic mice. Furthermore, Car@PLGA-NPs significantly decreased pro-inflammatory cytokines (IL-1β, IL-6, TNF-α) and increased the anti-inflammatory cytokine IL-10 (*P* < 0.001, **Figures 2E–H**). Treatment also reduced oxidative stress, evidenced by significantly decreased MDA levels and increased SOD activity (*P* < 0.001, **Figures 3A, B**). Immunostaining of pancreatic islets demonstrated that Car@PLGA-NPs effectively reversed diabetes-associated hormonal dysregulation: significantly increasing depleted insulin levels and decreasing elevated glucagon levels in db/db mice (*P* < 0.001, **Figures 3C, D**). Collectively, these data confirmed that Car@PLGA-NPs were superior to free carvacrol in modulating pancreatic α/β-cell hormones (glucagon/insulin), improving islet function, attenuating inflammation and oxidative stress, and ameliorating diabetic pathology.

### 3.3 Effect of Car@PLGA-NPs on the pathological structure of colon in mice

**Figure 4:**
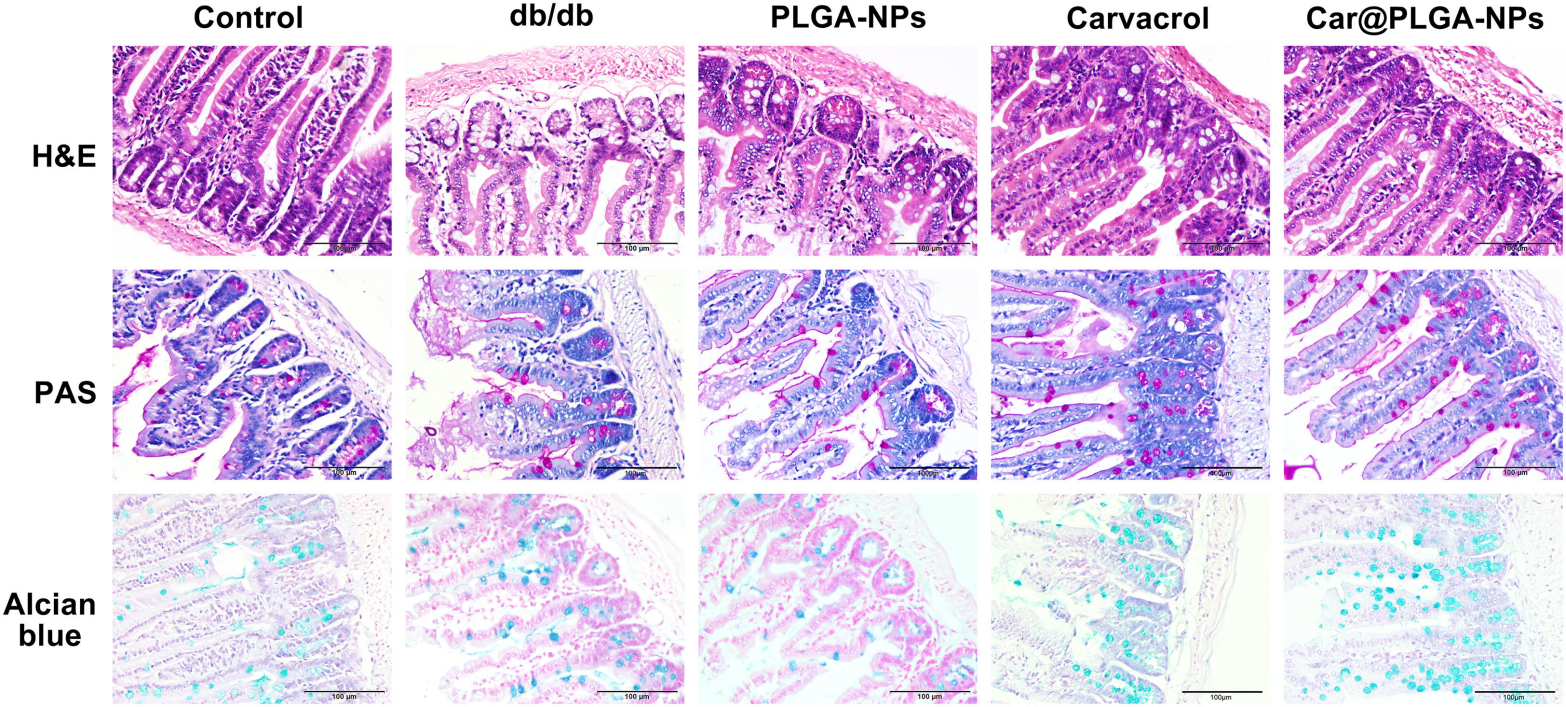
Car@PLGA-NPs relieves colon tissue damage. H&E, PAS and AB staining of colon tissue of mice in each group. Scale bar, 100μm.

Histopathological analysis of mouse colon tissues (H&E, PAS, and AB staining) revealed disrupted crypt architecture and inflammatory cell infiltration in db/db mice by H&E staining. PAS and AB staining further demonstrated significantly reduced goblet cell density and acidic mucin secretion areas in db/db mice colons. Both Car@PLGA-NPs and free carvacrol ameliorated these pathological alterations—reducing inflammatory infiltration, restoring crypt structure, and increasing goblet cell numbers and acidic mucin secretion areas. Notably, Car@PLGA-NPs exhibited more pronounced therapeutic efficacy than free carvacrol in reversing these changes (**Figure 4**).

### 3.4 Car@PLGA-NPs Restructure Gut Microbiota in Diabetic Mice

**Figure 5:**
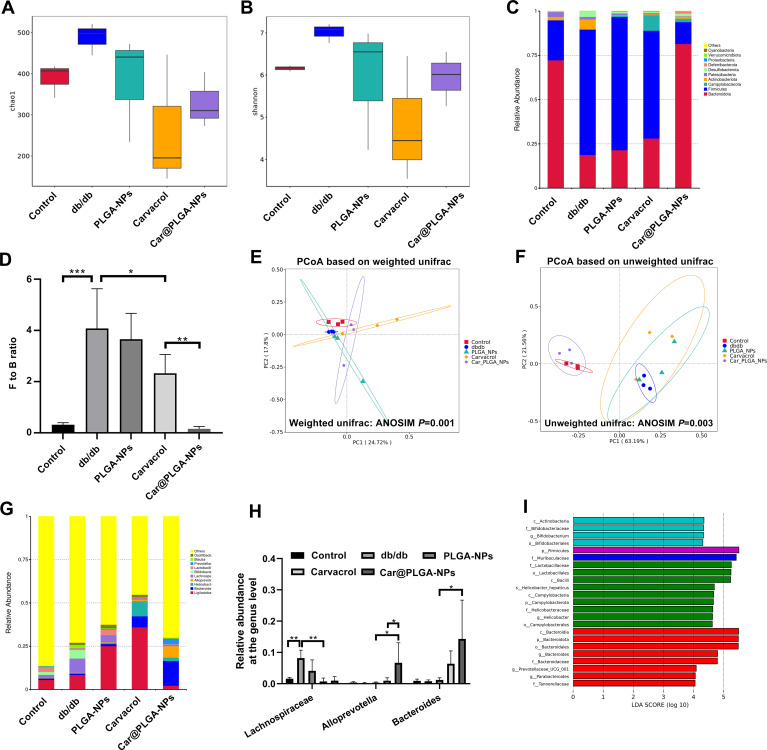
Analysis of gut microbiota by 16S rRNA sequencing. **(A)** chao index; **(B)** shannon index; **(C)** Histogram of relative species abundance at phylum level; **(D)** Ratio of Firmicutes to Bacteroidetes in each sample group; **(E, F)** PCoA analysis; **(G)** Histogram of relative species abundance at genus level;**(H)** Gut microbiota with significant differences at the genus level among sample groups; **(I)** Histogram of LDA value distribution obtained by LEfSe analysis; ^*^P < 0.05, ^**^P < 0.01, ^***^P < 0.001.

Based on the experimental results obtained from the above research, we concluded that both free Carvacrol and Car@PLGA-NPs could significantly improve the pathological structure of the colon in diabetic mice. Given that the integrity of intestinal morphology is closely related to the balance of the intestinal microbiota, we hypothesized that the intestinal protective effect of Carvacrol might be related to its regulation of the intestinal microbiota. Therefore, we conducted 16S rRNA sequencing on the feces of each group of mice. 16S rRNA sequencing of fecal DNA revealed significant gut microbiota alterations in diabetic mice: α-diversity (Shannon/Chao indices) increased in db/db mice versus controls (*P* < 0.05) but decreased following Car@PLGA-NPs or free carvacrol treatment (**Figures 5A, B**). Dominated by Bacteroidetes and Firmicutes at the phylum level (**Figure 5C**), db/db mice exhibited a markedly elevated Firmicutes-to-Bacteroidetes (F/B) ratio (*P* < 0.01 vs. controls). Crucially, Car@PLGA-NPs not only reversed this imbalance but achieved significantly lower F/B ratios than free carvacrol (*P* < 0.05, **Figure 5D**). We conducted principal coordinate analysis (PCoA) based on the Weighted unifrac distance and Unweighted unifrac distance to compare the microbial communities and compositions among different groups. Moreover, the similarity analysis (ANOSIM) indicated that there were statistically significant differences in the overall composition of the intestinal microbial communities among the groups (*P* < 0.01) (**Figures 5E, F**). The results of relative abundanceat the genus level showed: 1) In db/db mice, Lachnospiraceae significantly increased compared to the control group (*P* < 0.01), while Lachnospiraceae in db/db mice treated with free Carvacrol significantly decreased (P < 0.01); 2). In the Car@PLGA-NPs group, the abundances of *Bacteroides* and *Alloprevotella* were significantly increased compared to the PLGA-NPs group (*P* < 0.01), and the abundance of *Alloprevotella* was also significantly increased compared to the free Carvacrol group (*P* < 0.01) (**Figures 5G–I**). To investigate the correlations between gut microbiota and phenotypic indices, we performed correlation analyses of FBG, TG, TC, HDL-C, LDL-C, and serum insulin levels with three significantly changed bacterial genera identified from 16S rRNA sequencing (**Table 2**). The results revealed that Lachnospiraceae showed inverse correlations with the other indicators but a positive correlation with HDL-C. *Alloprevotella* and *Bacteroides* were negatively correlated with HDL-C and positively correlated with the remaining parameters. Notably, *Bacteroides* exhibited statistically significant correlations with all these indicators (*P* < 0.05), with the highest absolute correlation coefficients. These findings suggest that microbial alterations are associated with host metabolic improvements. Collectively, Car@PLGA-NPs uniquely remodeled gut microbial composition/abundance in diabetic mice, demonstrating superior modulatory efficacy over free carvacrol.

**Table 2:** Correlation analysis between gut microbiota (Lachnospiraceae*, Bacteroides, Alloprevotella*) and phenotypic indices (FBG、TG、TC、HDL-C、LDL-C and Serum insulin.

Factors	Correlation index	P value
Lachnospiraceae, FBG	-0.533	0.041
Lachnospiraceae, TG	-0.497	0.059
Lachnospiraceae, TC	-0.551	0.033
Lachnospiraceae, HDL-C	0.577	0.024
Lachnospiraceae, LDL-C	-0.552	0.033
Lachnospiraceae, Serum insulin	-0.539	0.038
*Bacteroides*, FBG	0.768	0.001
Bacteroides, TG	0.609	0.016
Bacteroides, TC	0.720	0.002
Bacteroides, HDL-C	-0.704	0.003
Bacteroides, LDL-C	0.670	0.006
Bacteroides, Serum insulin	0.719	0.003
*Alloprevotella*,FBG	0.591	0.020
*Alloprevotella*, TG	0.393	0.148
*Alloprevotella*, TC	0.554	0.032
*Alloprevotella*, HDL-C	-0.533	0.041
*Alloprevotella*, LDL-C	0.479	0.071
*Alloprevotella*, Serum insulin	0.530	0.042

### 3.5 Car@PLGA-NPs Modulate Endoplasmic Reticulum Stress Signaling

**Figure 6:**
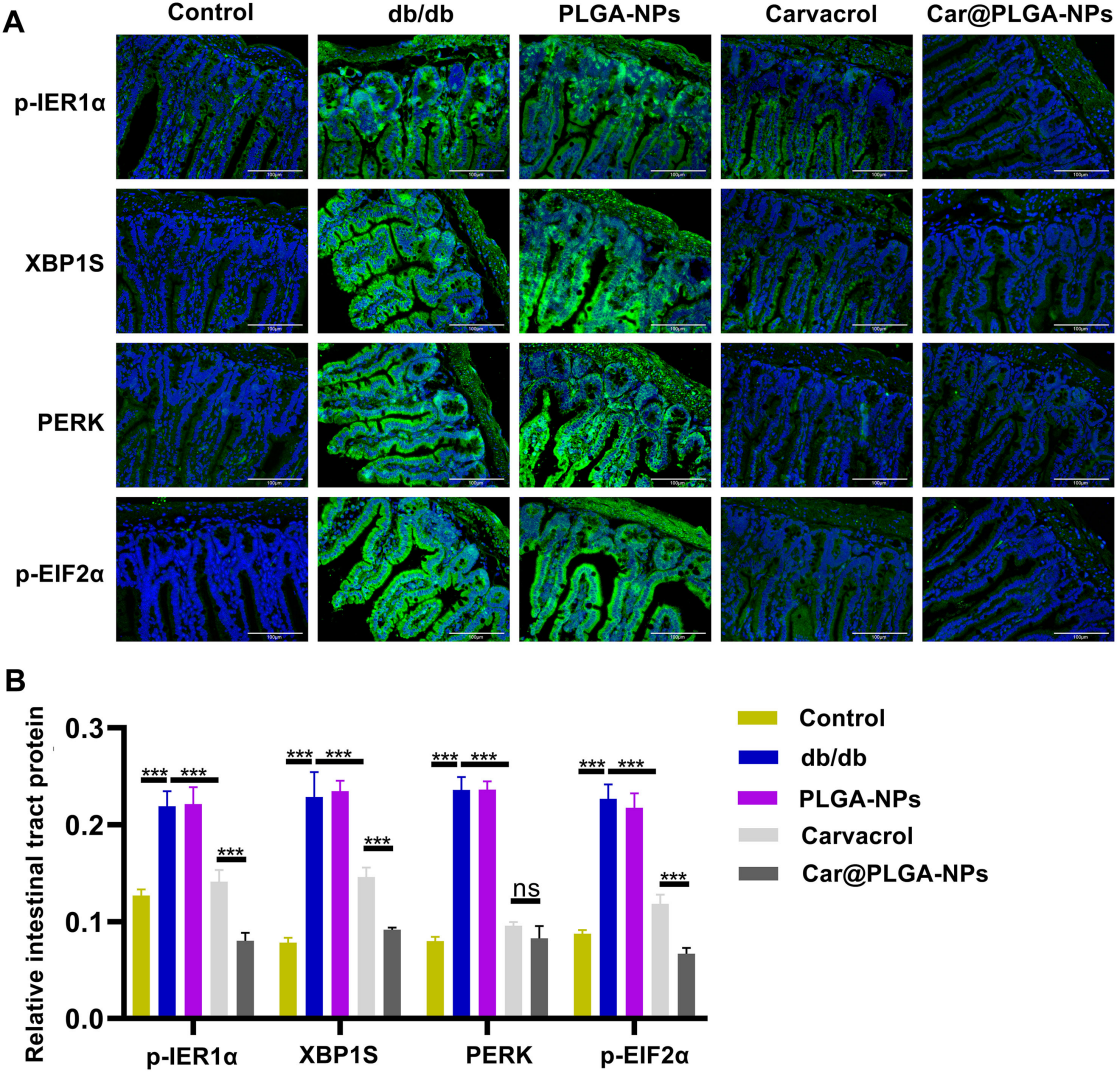
Expression of endoplasmic reticulum stress factors in mice intestinal tissues. **(A)** The expressions of p-IRE1α, XBP1S, PERK and p-ElF2α in intestinal tissues of mice in each group were detected by immunofluorescence, Scale bar, 100μm; **(B)** Quantification of fluorescence intensity. ^***^P < 0.001, ns, none significance.

**Figure 7:**
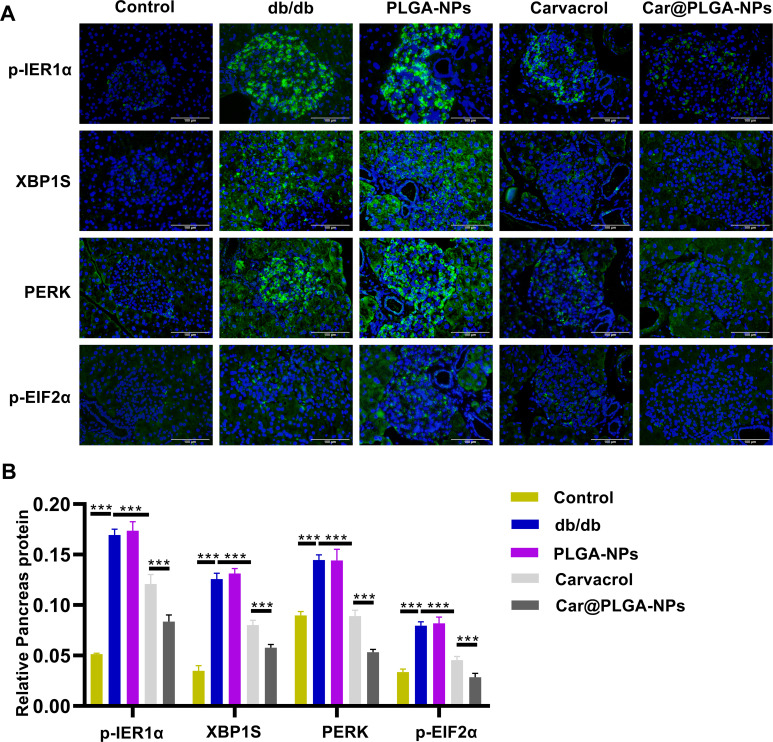
Expression of endoplasmic reticulum stress factors in mice Pancreas tissues. **(A)** The expressions of p-IRE1α, XBP1S, PERK and p-ElF2α in Pancreas tissues of mice in each group were detected by immunofluorescence, Scale bar, 100μm; **(B)** Quantification of fluorescence intensity. ***P<0.001.

**Figure 8:**
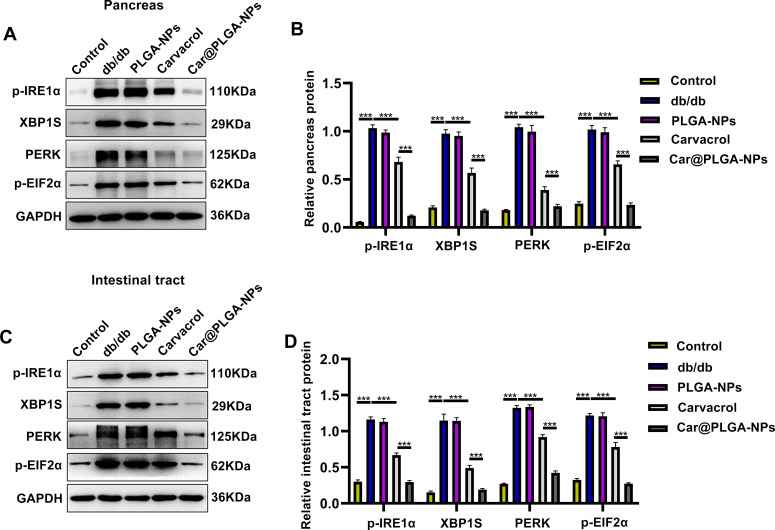
Expression of endoplasmic reticulum stress factors in pancreas and intestinal tract. **(A, B)** The expressions of p-IRE1α, XBP1S, PERK and p-ElF2α in pancreas were detected by Western blot. **(C, D)** The expressions of p-IRE1α, XBP1S, PERK and p-ElF2α in intestinal tract were detected by Western blot. ***P<0.001.

The gut microbiota can influence the cellular stress state through the circulatory system, including regulating the endoplasmic reticulum stress pathway (He et al., 2025). Based on the significant regulatory effects of free Carvacrol and Car@PLGA-NPs on the gut microbiota that we observed in Section 3.4, we will next further explore the mechanism by which Carvacrol improves the functions of the pancreas and colon by detecting the expression of key markers of endoplasmic reticulum stress. We assessed the expression of endoplasmic reticulum (ER) stress-related proteins (p-IRE1α, XBP1S, PERK, and p-ElF2α) in pancreatic and colonic tissues using immunofluorescence (**Figures 6** and **7**) and Western blotting (WB) (**Figure 8**). Compared to the db/db group, treatment with free carvacrol and Car@PLGA-NPs significantly reduced the expression levels of these ER stress proteins in both tissues of db/db mice. Notably, Car@PLGA-NPs suppressed ER stress protein expression more effectively than an equivalent dose of free carvacrol, highlighting the advantage of the nano-formulation (*P* < 0.001, **Figures 6** and **8**). To further validate the correlation between gut microbiota changes and ER stress protein expression, we performed Spearman correlation analysis between the expression levels of p-IRE1α, XBP1S, PERK, and p-ElF2α and three significantly altered species identified from the 16S rRNA sequencing results. The Lachnospiraceae is positively correlated with the endoplasmic reticulum stress-related proteins, while *Bacteroides* is negatively correlated with these proteins. *Alloprevotella* is also negatively correlated with the endoplasmic reticulum stress-related proteins, especially showing a significant negative correlation with the levels of p-IRE1α and p-EIF2α (*P* < 0.01). As a type of intestinal microorganism, the higher the abundance of *Alloprevotella*, the lower the cellular stress state (manifested by the decreased levels of p-IRE1α and p-EIF2α), which may represent a beneficial protective effect (**Table 3**). These data suggest that the protective effect of Car@PLGA-NPs may be related to its ability to simultaneously regulate the intestinal microbiota and inhibit endoplasmic reticulum stress. The significant correlation between these two factors indicates a potential ‘intestinal-organ axis’ mechanism.

**Table 3:** Correlation analysis between gut microbiota species (*Lachnospiraceae*, *Bacteroides*, *Alloprevotella*) and the expression of p-IRE1α, XBP1S, PERK, and p-ElF2α Proteins.

Factors	Correlation index	P value
*Lachnospiraceae*, p-IRE1α	0.339	0.216
*Lachnospiraceae*, XBP1S	0.461	0.084
*Lachnospiraceae*, PERK	0.446	0.095
*Lachnospiraceae*, p-ElF2α	0.414	0.125
*Bacteroides*, p-IRE1α	-0.504	0.056
*Bacteroides*, XBP1S	-0.293	0.289
*Bacteroides*, PERK	-0.332	0.226
*Bacteroides*, p-ElF2α	-0.429	0.111
*Alloprevotella*, p-IRE1α	-0.571	0.026
*Alloprevotella*, XBP1S	-0.425	0.114
*Alloprevotella*, PERK	-0.436	0.104
*Alloprevotella* , p-ElF2α	-0.600	0.018

## 4 Discussion

Type 2 diabetes mellitus (T2DM) arises from insulin resistance, characterized by reduced target organ sensitivity to insulin, coupled with insufficient pancreatic beta-cell insulin secretory capacity, collectively leading to hyperglycemia (Bielka et al., 2022). Contemporary treatment strategies extend beyond glycemic control to address associated metabolic dysregulations (Lu et al., 2024). Natural compounds like carvacrol offer therapeutic potential due to their efficacy and favorable safety profile (Hoca et al., 2024). To enhance carvacrol’s duration of action, bioavailability, and effectiveness, this study developed Carvacrol-Loaded Poly Nanoparticles (Car@PLGA-NPs) and evaluated them in db/db mice. Our study successfully developed a novel nano-formulation of carvacrol, Car@PLGA-NPs, which demonstrated superior therapeutic efficacy over its free form in a db/db mice model of T2DM. The enhanced bioavailability and sustained release profile of Car@PLGA-NPs underpinned their robust multi-faceted effects, including amelioration of dyslipidemia, systemic inflammation, oxidative stress, and pancreatic islet dysfunction. More importantly, our investigation moves beyond the established anti-inflammatory and antioxidant properties of carvacrol to propose a novel mechanistic axis: the amelioration of diabetic symptoms is mediated through concurrent remodeling of the gut microbiota and suppression of endoplasmic reticulum (ER) stress in both pancreatic and intestinal tissues. This proposed “gut microbiota-ER stress axis” elucidates a novel mechanistic pathway and represents a significant advancement in understanding how carvacrol, particularly in its nano-formulated state, exerts its therapeutic benefits.

T2DM is characterized by insulin resistance, hyperglycemia, and dyslipidemia (Jaishree and Narsimha, 2020), with oxidative stress being a key contributor to its complications, as reflected by alterations in MDA levels and SOD activity (Xie et al., 2021). Previous studies have shown that the combination of free carvacrol with rosiglitazone/thiazolidinediones improves blood glucose and insulin levels in T2DM mice (Hoca et al., 2024). This study found that in the db/db mice model, carvacrol alone effectively regulated blood glucose, insulin, and lipid parameters, which aligns with existing reports. Critically, Car@PLGA-NPs significantly outperform free carvacrol in regulating pancreatic α/β-cell hormones (glucagon/insulin) and improving islet function. While the improved pharmacokinetics (e.g., sustained release and enhanced bioavailability) conferred by the nano-formulation likely contributed to this superiority, the *in vivo* outcomes suggest an additional or synergistic mechanism. The PLGA-based delivery system appears to not only preserve but also amplify carvacrol’s intrinsic bioactivities, such as its potential anti-inflammatory action via the TLR4/NF-κB pathway (Zhao et al., 2021). This may be due to enhanced tissue targeting or intracellular uptake, leading to a more substantial restoration of α/β-cell hormonal balance. The concurrent and pronounced amelioration of oxidative stress further underscores the multi-targeted potential of this nano-therapeutic strategy.

The progression of T2DM is associated with distinct alterations in the gut microbiota, underscoring complex host-microbiota interactions (Zhou et al., 2022). Such dysbiosis can compromise intestinal epithelial integrity, leading to excessive release of lipopolysaccharide (LPS) from Gram-negative bacteria, which subsequently disrupts the intestinal mechanical barrier and tight junction function (Stephens and von der Weid, 2020; Hua et al., 2022). Notably, monomeric compounds from traditional Chinese medicine (TCM), like carvacrol, have demonstrated the capacity to restore microbial homeostasis under pathology by modulating microbial abundance and alleviating intestinal inflammation, as evidenced in LPS-induced models (Xu et al., 2013; Yang et al., 2017; Feng et al., 2019). Therefore, targeting the gut microbiota represents a promising therapeutic strategy for T2DM. Previous research has indicated that the Firmicutes*/*Bacteroidetes (F/B) ratio is elevated in patients with T2D compared to healthy individuals (Bahar-Tokman et al., 2022). Consistent with this, our findings demonstrate that Car@PLGA-NPs significantly reduced the F/B ratio in db/db mice. An increase in Lachnoclostridium abundance contributes to inflammation and insulin resistance (Zhang et al., 2020). *Alloprevotella* is often associated with fiber degradation and SCFA production. Moreover, an increase in its abundance alleviates inflammation and is closely linked to the treatment of T2DM (Yin et al., 2024). In this study, Car@PLGA-NPs significantly reduced the abundance of Lachnospiraceae while increasing that of *Alloprevotella*. Furthermore, the correlation between gut microbiota and phenotypic indices that microbial alterations are associated with improved host metabolism. Interestingly, although db/db mice exhibited increased gut microbiota α-diversity compared to control mice, we hypothesize that this may reflect an overgrowth of pathogenic or opportunistic species, ultimately resulting in a dysbiotic state. Car@PLGA-NPs counteracted this shift, supporting a more balanced microbial structure. Moreover, the anti-inflammatory and antioxidant effects of Car@PLGA-NPs may also involve modulation of Bacteroidetes, a hypothesis that will be further tested in future microbiota transplantation studies. In summary, our findings indicate that Car@PLGA-NPs exert multi-faceted therapeutic effects in diabetic mice by remodeling the gut microbiota, improving islet function, and enhancing colonic barrier integrity. These insights offer a novel strategic perspective for the intervention of T2DM.Endoplasmic reticulum stress (ERS) is closely linked to the onset and progression of diabetes. In pancreatic β-cells, chronic hyperglycemia and lipotoxicity exacerbate ERS, thereby activating the unfolded protein response (UPR), which ultimately triggers apoptosis and reduces β-cell mass (Dekkers et al., 2024). ERS involves alterations in multiple signaling pathways: the IRE1α-XBP1 axis regulates ER homeostasis, while the PERK-ElF2α pathway primarily suppresses global protein translation; PERK activation further phosphorylates ElF2α to reduce the expression of associated proteins, and excessive activation of the IRE1α-XBP1S signal induces β-cell apoptosis (Lee and Lee, 2022). Some studies have shown that Carvacrol can alleviate ERS by inhibiting the expression of proteins related to the UPR pathways such as PERK and IRE1α (Evyapan et al., 2024; Yang et al., 2025). This is consistent with the research results of this experiment. This study demonstrates that Car@PLGA-NPs significantly downregulate the expression of p-IRE1α, XBP1S, PERK, and p-eIF2α in both pancreatic islet and colon tissues of db/db mice, indicating that the nanoformulation effectively and simultaneously suppresses the two core ERS pathways—IRE1α and PERK. This dual inhibitory effect holds important biological implications: on one hand, it alleviates the endoplasmic reticulum protein-folding burden in β-cells, thereby helping to preserve their function; on the other hand, it repairs intestinal epithelial barrier damage induced by ERS, thus interrupting the vicious cycle between inflammation and metabolic dysfunction. These results fully illustrate that the Car@PLGA-NPs delivery system, by enhancing the bioavailability of carvacrol, achieves maximal therapeutic benefits in the diabetic model through multi-tissue protection and metabolic improvement.

In the intestinal environment, ERS is also recognized as a contributor to inflammation development, which impairs intestinal epithelial barrier function and is associated with diseases like inflammatory bowel disease (IBD) (Fan et al., 2024). Furthermore, several significantly altered species were identified within the intestinal microbiota of Car@PLGA-NP-treated db/db mice. Correlation analysis indicated that Car@PLGA-NP-induced enrichment of *Alloprevotella* was negatively correlated with colonic ER stress. Based on these correlative findings, we hypothesize that a key mechanism of Car@PLGA-NPs involves the remodeling of the gut microbiota, particularly by enriching beneficial short-chain fatty acid (SCFA)-producing bacteria such as *Alloprevotella*. This microbial restoration enhances intestinal barrier integrity and reduces the infiltration of inflammatory molecules, thereby alleviating the metabolic and inflammatory burden on distal organs. The subsequent decline in systemic stress signals further mitigates the endoplasmic reticulum protein-folding load in pancreatic β-cells and intestinal enterocytes. Thus, the gut microbiota serves as an upstream regulator of tissue ER stress, and Car@PLGA-NPs therapeutically target the entire “gut microbiota–ER stress axis”.

In conclusion, this study establishes Car@PLGA-NPs as a highly effective nano-therapeutic strategy for T2DM. Its innovation lies not only in the enhanced delivery of carvacrol but also in the elucidation of a novel multi-organ mechanistic axis underlying its efficacy. Our findings suggest that the gut microbiota can systemically influence ER stress in critical metabolic tissues provide a new conceptual framework for understanding the pathophysiology of diabetes and the action of natural product-based therapeutics. While this correlative evidence is strong, future studies employing antibiotic treatment, fecal microbiota transplantation, or germ-free models are warranted to establish a definitive causal relationship within this axis. This work lays a solid foundation for the development of microbiota-targeting nanomedicines for metabolic diseases.

## Data Availability

The datasets presented in this study can be found in online repositories. The names of the repository/repositories and accession number(s) can be found in the article/supplementary material.
